# Pulmonary sequestration associated with pulmonary actinomycosis: A case report and literature review

**DOI:** 10.1097/MD.0000000000039981

**Published:** 2024-10-04

**Authors:** Yun Wang, Guangfeng Ma, Na-na Rao, Mubo Liu, Jiangrong Liao, Qian-yu Wang

**Affiliations:** a Respiratory Department of Guizhou Aerospace Hospital affiliated with Zunyi Medical University, Zunyi, China; b Central Laboratory of Guizhou Aerospace Hospital affiliated with Zunyi Medical University, Zunyi, China.

**Keywords:** penicillin, pulmonary actinomycosis, pulmonary sequestration, surgical treatment

## Abstract

**Rationale:**

Pulmonary sequestration (PS), a rare pulmonary disease, arises from congenital pulmonary vascular dysplasia. Meanwhile, pulmonary actinomycosis is a purulent infection of lung lesions triggered by the inhalation of actinomycetes, which is also uncommon. Even rarer is the occurrence of pulmonary actinomycete infection secondary to PS. Herein, we present a case report of such a rare occurrence.

**Patient concerns:**

The 21-year-old female patient had been erroneously diagnosed with pneumonia and tuberculosis, presenting symptoms of cough, sputum, and hemoptysis; however, the implemented anti-infection and antituberculosis treatments proved to be ineffective.

**Diagnoses:**

The diagnosis of the sequestration in the right lower lung was confirmed through an enhanced chest CT scan and a 3-dimensional reconstruction of the pulmonary vessels.

**Interventions:**

During the surgical video-assisted thoracoscopic resection of the right lower lobe lesion, it was discovered that the isolated lung tissue’s blood supply vessel originated from the thoracic aorta. Additionally, the pathological examination revealed that the lung tissue of the right lower lobe lesion was infected with pulmonary actinomycetes.

**Outcomes:**

Following thorough evaluation, the patient received a final diagnosis of pulmonary actinomycete infection that occurred secondary to right lower lung sequestration. Consequently, they underwent treatment consisting of high-dose penicillin administered for a period of 6 months post-operation. Over the course of the subsequent 23-month follow-up, no recurrence of the infection or abnormal CT scan findings were observed.

**Lessons:**

Pulmonary sequestration bears clinical resemblance to pulmonary actinomycetes. In cases where recurrent episodes of pneumonia occur at the same location, and chest imaging indicates persistent lesions in the basal segment of the lower lobe near the spine, the possibility of PS should be considered. Prompt chest-enhanced CT and 3-dimensional reconstruction of pulmonary vessels are crucial for a definitive diagnosis. Imaging findings such as mass-like consolidation, cystic lesions, liquefactive necrosis, and pneumonia-like changes, coupled with typical air suspension signs and sulfur-like particles visible under tracheoscopy, suggest a potential pulmonary actinomycete infection. Timely biopsy is essential to confirm the diagnosis and prevent missed or incorrect diagnoses.

## 1. Introduction

Pulmonary sequestration (PS), also known as bronchopulmonary sequestration, is a rare lung disease in which, during embryonic development, the pulmonary artery hypoplasia renders a portion of the lung tissue with a disturbed blood supply and replaced by a branch of the aorta with the pulmonary artery supply area, the lung tissue, which is unable to undergo normal oxygenation because the oxygen content of the blood from the aorta is quite different from that from the blood from the pulmonary artery, And thus hypoplasia, which leads to no lung function in that segment of lung tissue.

## 2. Case report

General clinical data: A 21-year-old female patient, a farmer, unmarried, was admitted to our hospital on February 03,2022 due to recurrent cough and expectoration with hemoptysis for 2 years and 3 days for the current episode. She experienced cough and expectoration 2 years ago after exposure to cold weather. The cough was paroxysmal and accompanied by wheezing after physical activity. She also had intermittent hemoptysis but no night sweats, chills, fever, or chest pain. She visited a local county hospital where she was diagnosed with pneumonia and showed improvement after treatment. Cough, expectoration, and hemoptysis reappeared 1 year ago, and she sought medical attention at a local people’s hospital where she was diagnosed with pulmonary tuberculosis. She received antituberculosis treatment until this admission, which led to an improvement in her cough and hemoptysis. Three days prior to admission, she experienced another episode of cough and hemoptysis after exposure to cold weather. The hemoptysis was intermittent, bright red in color, and occurred

**Figure 1.**
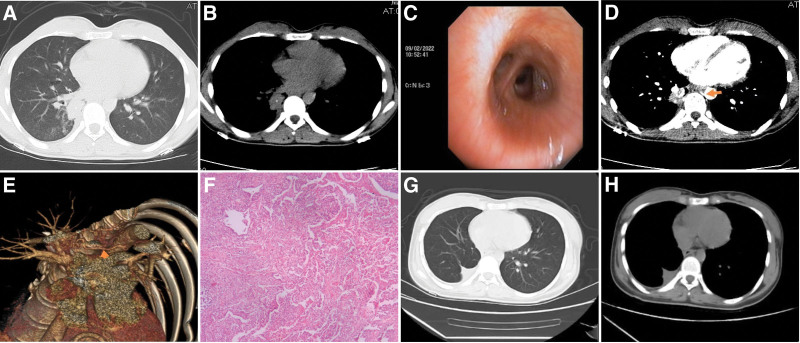
(A, B) The chest CT scan revealed a dense mass-like shadow in the basal segment of the right lower lobe of the lung near the paraspinal column, accompanied by foci of calcification. (C) Bronchoscopy identified congestion and the presence of yellow mucous sputum adhering to the opening of the right lower basal segment. The examination did not reveal any stenotic neoplasms or obstructions within the lumen. (D, E) Enhanced CT of the chest, along with a 3-dimensional reconstruction of the pulmonary vasculature, demonstrated that the arterial blood supply to the right lower basal segment of the lung originated from the thoracic aorta (arrows indicate the abnormal arteries supplying the blood). (F) The postoperative pathology of the right lower lung lesion showed neutrophilic granulocyte exudation in the alveolar lumen, bronchioles, and fine bronchial lumens. The bronchial lumens exhibited radially arranged hyphae, with the tips of the hyphae expanding in a pestle and mortar-like pattern. Some hyphae appeared wrapped up in a mass, consistent with actinomycosis infection. (G, H) A follow-up chest CT on January 17, 2024, revealed postoperative changes in the right lower lung field, with the condition showing stability.

Three to five times a day, with 10 to 20 mL of blood each time. The previous treatment at the local hospital had suboptimal results, prompting her to seek further diagnosis and treatment at our hospital. Her psychiatric condition, eating, and sleeping patterns were normal. Her bowel movements were regular, and she did not report any significant weight changes. She had a history of alcohol consumption prior to February but denied smoking or any other specific vices. Menstruation was normal, and she denied having any other chronic diseases, infectious diseases, or family genetic disorders. Physical examination and auxiliary tests revealed the following findings: body temperature was 37.1 °C, pulse rate was 86 beats per minute, respiration rate was 16 beats per minute, blood pressure was 120/66 mm Hg, transcutaneous finger oxygen saturation was 96% without supplemental oxygen. The skin and sclera appeared normal without any yellow discoloration. There were no palpable or enlarged superficial lymph nodes throughout the body. The oral mucosa was smooth, gingiva was nonerythematous and without purulent discharge. Dental examination showed evidence of no caries, dentures, missing teeth, and residual roots. Jugular vein was not distended and there was no evidence of hepatic jugular venous return. The thorax appeared symmetrical with no widened intercostal spaces. Bilateral respiratory motion and thoracic expansion were symmetrical. There was no enhancement or reduction in double lung voice tremor. Breath sounds were clear bilaterally, with wet rales heard in the right lower lung but no wheezing. Heart rate was 86 beats per minute, with no arrhythmia or abnormal abdominal signs. There was no edema observed in both Lower limbs. Blood routine examination revealed a white blood cell count of 6.21 × 10^9^/L, with neutrophils at 5.1 × 10^9^/L and lymphocytes at 5.1 × 10^9^/L. Red blood cell count was 4.02 × 10^12^/L, hemoglobin level was 125 g/L, and platelets measured 213 × 10^9^/L. Liver and kidney function tests, as well as hematuria, blood glucose, electrolytes, coagulation function, stool routine, acid-fast staining of sputum (performed 3 times), and nucleic acid DNA testing for Mycobacterium tuberculosis in sputum all yielded normal results. Sputum culture over a period of 5 days did not show any fungal growth. Measurement of D-D polymer levels was 1.02 µg/mL, while blood fungal and GM examinations yielded negative results. Sputum culture revealed normal flora growth and no fungal spores or hyphae were detected on sputum smear. Tuberculosis antibody test was negative, Mycoplasma pneumoniae antibody levels were within normal range. Human immunodeficiency virus, hepatitis B surface antigen, and syphilis antibody tests were negative. Tuberculosis infection T cell test was negative. Erythrocyte sedimentation rate was 50 mm/h and C-reactive protein level was 20.6 mg/L. Chest CT plain scan showed patchy, nodular dense shadow with increased density in the lower lobe of the right lung, suggesting possible infectious lesions or space-occupying lesions to be evaluated further (shown in Fig. 1A and B).

Diagnosis and Treatment after Admission: Following admission, the patient received treatment for anti-ceftazidime infection, cough, hemostasis, and symptomatic relief. Tracheoscopy was performed, revealing congestion and yellow mucopurulent attachment in the opening of the right lower lobe basal segment. No stenosis, new organisms, or obstruction were observed in the lumen (shown in Fig. 1C). Additionally, alveolar lavage and brushing examination were carried out in the outer posterior basal segment of the right lower lobe, showing negative results for acid-fast staining in both tracheoscopic lavage fluid and brush examination. Furthermore, the alveolar lavage fluid showed no presence of respiratory etiological nucleic acid DNA. Fungal and general bacterial cultures, as well as dislodged cell checks, yielded negative results. The lavage fluid Xpert test also came back negative. Given the relatively shaped lesions in the right lower lung, the possibility of neoplastic lesions could not be ruled out. Therefore, chest-enhanced CT and 3-dimensional reconstruction of pulmonary blood vessels were conducted, indicating abnormal feeding arteries originating from the thoracic aorta in the lesions of the lower lobe of the right lung. A diagnosis of PS in the lower lobe of the right lung, exhibiting calcification focal, was made (shown in Fig. 1D and E). With this diagnosis, the presence of right lower lung PS was confirmed. On February 17, 2022, following the administration of general anesthesia, a resection of the right lower lobe lesion was carried out with the aid of surgical thoracoscopy. During the surgical procedure, adhesion was observed between the right lower lung and the pleura. Utilizing a high-frequency electric knife, the adherent area between the right lower lung and the chest wall was cauterized, revealing a mass approximately 4 cm in diameter in the lower lobe of the right lung. An abnormal blood supply artery was identified and isolated. This abnormal artery originated from the thoracic aorta, located near the front of the esophagus, and measured approximately 0.8 cm in diameter. After ligation, the artery was successfully disconnected. Subsequently, the right lower pulmonary artery, right lower lobe bronchus, and right lower pulmonary vein were gradually isolated and disconnected, allowing for the complete excision of the right lower lung lesion. Upon excision, the specimen revealed a mass measuring approximately 5 × 4 cm, containing a milky white jelly-like substance. The excised tissue was sent for pathological examination, which indicated a significant neutrophil exudate in the alveolar cavity, bronchus, and bronchiolar lumen. Additionally, actinomycetes were detected in the bronchial lumen, corroborating a diagnosis of pulmonary actinomycete infection (shown in Fig. 1F) indicating a secondary infection resulting from PS. Following the completion of surgery, the patient received penicillin intravenous drip of 10 million U bid for 12 days, leading to a significant improvement and subsequent discharge. Following discharge, the patient continued treatment with oral Amoxicillin Capsules (1 g; 3 times a day). A follow-up chest CT on January 17, 2024, revealed a stable condition (shown in Fig. 1G and H). The patient had no respiratory symptoms during the follow-up period.

## 3. Discussion

Pulmonary sequestration is an uncommon clinical condition and a congenital lung developmental anomaly that represents around 0.15% to 6.4% of lung diseases.^[1]^ It is primarily caused by a branch of the aorta replacing the pulmonary artery to supply the local lung tissue. This tissue is dysplastic due to the significant difference in oxygen content between the blood from the aorta and that from the pulmonary artery. Consequently, the lung tissue segment is unable to undergo normal oxygenation. The diagnostic standard characterizing this condition is the presence or absence of an intact visceral pleura.^[2]^ The prevailing theory regarding the origin of PS is the tractional doctrine, although the theory of pulmonary hypoplasia also holds some merit. Among these theories, the tractional doctrine is widely accepted in clinical practice.^[3,4]^ PS can be categorized into intralobar and extralobar types, with the intralobar type being the most common, accounting for approximately 75% of cases.^[5]^

Intralobar type lung sequestration is characterized by the same pleural membrane enveloping adjacent normal lung tissue, some of which may be solid lung tissue. The demarcation between normal lung tissue and the sequestered segment is often unclear. The sac enclosing the sequestered tissue is filled with mucus and generally does not communicate with normal bronchi.^[6]^ However, it may establish communication with adjacent bronchi following infection, leading to the presence of pus in the sac, and allowing air to enter. Paraspinal region at the posterior basal segment of the lower lobe of the left lung is the most common location for intralobar lung sequestration, with around 2/3 of cases found in the paraspinal groove at this segment of either the left or right lower lobe. On the other hand, the extralobar pattern is encased by a separate pleura, and the affected tissue is predominantly nonfunctional solid lung tissue. In some instances, this tissue may exhibit cystic changes, but it is generally not prone to infection. This pattern is commonly observed between the lower lobes of the lung and the diaphragm, with occasional occurrences below the diaphragm or within the mediastinum.

The main blood vessels of the isolated lung originate from branches of the systemic circulation. They primarily pass through the inferior pulmonary ligament to reach the affected area (after traversing the ipsi-lateral inferior pulmonary artery). The majority of these feeding arteries arise from a single source, although some may have 2 or even 3 origins. These sources include the thoracic aorta, abdominal aorta, celiac artery and its branches, subclavian artery, and intercostal arteries.^[7]^ There have also been reports of feeding arteries arising from the inferior vena cava, coronary arteries, right internal mammary artery, and renal arteries.^[8–11]^ The supply arteries exhibit varying thickness, with certain ones even measuring up to 1 cm in diameter. The venous return from the pulmonary isolation was inconsistent, leading to reflux of blood from the pulmonary isolation in the lobar type into the lower pulmonary vein. Consequently, this results in left to left shunting.

In addition, blood reflux from the lobe type lung can also occur into the semiazygous vein, azygous vein, inferior vena cava, innominate vein, intercostal vein, and others. PS is more symptomatic in adolescents aged 10 to 40 years, particularly in males compared to females. The intralobar type is more common than the extralobar type, with a left predominance. The classification and clinical manifestations of PS can significantly differ. Common symptoms include fever, cough, expectoration, chest pain, bloody sputum, and hemoptysis. The location of the lesion is prone to recurrent infections, which can be reduced through treatment, but still susceptible to recurring attacks. Chest radiographs can isolate intralobar type lungs, revealing dense and uniformly thickened shadows in the inner and posterior basal segments of the lower lobes closely adjacent to the diaphragmatic surface. The shape of the mass is typically round or ovoid, although occasionally triangular or polygonal. The borders are generally clear, with their long axes pointing posteriorly, suggesting a connection with descending arteries. If there is a connection with a bronchus, it appears as a single or multiple fluid-filled round shadow resembling a lung cyst.

The thickness of the cyst wall varies, surrounded by inflammatory changes in imaging. The size of the shadow can change during the course of the disease, increasing during infection and shrinking after absorption of inflammation, but it does not completely disappear. In some cases, the lesions in the posterior segment of the lower lobes may overlap with the shadows of the spine or heart on plain chest radiography. However, chest CT can provide a clear visualization of the shape, contour, and internal structure of the lesions. The lesions typically have well-defined borders, appearing round, oval, or triangular and may exhibit varying cystic changes in size. Abnormal vascular imaging, such as a connection with the aortic shadow, may sometimes be observed on CT, presenting as a teased or striped shadow. The caudal end of the comma-shaped shadow represents abnormal arterial orientation. Three-dimensional imaging of the pulmonary artery is diagnostic for PS,^[12]^ with enhanced CT being the preferred examination method at present. Magnetic resonance imaging (MRI) also holds diagnostic value, with the ability to detect clear masses and their internal structures within the thoracic cavity. MRA can identify abnormal lung tissue and its relationship to surrounding organs, revealing abnormal arterial origin, course, and venous return.^[13]^

The current treatment options for PS include conservative, surgical, and interventional approaches. Some researchers suggest that conservative treatment may be effective for extralobar PS in asymptomatic individuals.^[14]^ However, for intralobar PS, lobectomy is commonly performed. With advancements in endoscopic technology, thoracoscopic lobectomy has become the primary treatment method, as it has been proven to result in lower complications and shorter hospital stays than conventional thoracotomy.^[15]^ In cases of PS accompanied by massive hemoptysis, some experts argue that interventional therapy should precede surgical treatment. This involves isolating the abnormal blood vessels responsible for the lung condition through embolization. Consequently, the isolated lung tissue undergoes denaturing and shrinking due to ischemia and absorption.^[16]^ When considering the appropriate treatment approach, it is essential to take into account various factors such as the patient’s physical function status, clinical symptoms, coexisting medical conditions, and their personal preferences.

Actinomycosis refers to a class of gram-positive bacteria characterized by their branching hyphae. The name is derived from their radial growth on solid media. The key members of this class include Actinomyces tunica, Actinomyces maltophilia, Actinomyces caries, Actinomyces vinculin, and Actinomyces sularis.^[17]^ It is worth noting that most actinomycetes possess mycelia that resemble fungi both in morphology and in growth and development. Consequently, they were previously classified as fungi.^[18]^ Aerobic actinomycetes constitute a group of microorganisms that strictly require oxygen for growth. These microorganisms tend to have a relatively slow growth rate. In terms of their pathogenicity to humans, actinomycetes can be classified into 2 groups based on the presence or absence of mycolic acid.^[19]^

The primary bacteria responsible for causing pulmonary actinomycosis are actinomycetes. These bacteria commonly inhabit various parts of the human oral cavity such as the oral mucosa, gingiva, tonsils, and colon. When the body’s immune system weakens, they can invade the respiratory tract through the aspiration of oral secretions. Initially, these bacteria cause lesions in the bronchus and subsequently invade the lung parenchyma. Moreover, they can also spread to the mediastinum through esophageal lesions, or the diaphragm and pleura through abdominal infections, leading to septic pneumonia in the lungs. In addition, they can invade the transpulmonary space and chest wall, forming sinus tracts in the ribs.^[20]^ Furthermore, these bacteria may also enter the bloodstream, causing systematic dissemination.^[21]^

Pulmonary actinomycosis typically has a gradual onset, characterized by mild symptoms such as low-grade or irregular fever, cough, and production of a small amount of mucoid sputum. However, as the disease progresses and multiple abscesses form in the lungs, the symptoms worsen. This can include high fever, excessive coughing, large amounts of mucopurulent sputum, and the presence of blood or massive hemoptysis in the sputum. The patient may also experience fatigue, night sweats, anemia, and weight loss. It is important to note that these symptoms can often be mistaken for tuberculosis,^[22]^ as well as other conditions such as lung tumors, lung abscesses, and pulmonary fungal infections. Consequently, pulmonary actinomycosis is sometimes referred to as “the great mimicker”.^[23]^” Pleural involvement by actinomycetes can result in severe chest pain, as well as the development of pleural effusion and empyema. In some cases, there may also be swelling in the face and upper extremities due to upper vena cava obstruction syndrome, or the presence of pericardial effusion.^[24]^ When the disease invades the chest wall, it may lead to the formation of subcutaneous abscesses and fistulas. These fistulas can discharge pus mixed with bacteria and cause pigmentation in the surrounding tissue.^[25]^ Even after the fistulas have healed, new fistulas may appear nearby. If the mediastinum is affected, it can result in respiratory issues or difficulty swallowing, and in severe cases, it can potentially be fatal.

The radiographic findings of pulmonary actinomycosis are nonspecific. The main findings on CT primarily include mass-like consolidation, cystic lesions, liquefactive necrosis, and pneumonia-like changes. Peripheral enhancement “air sign” in hypodense areas and pleural thickening are the more typical CT features of pulmonary actinomycosis.^[26]^ Early infection can present as a nodule with ill-defined peripheral borders in the lung, sometimes with internal interlobular septal thickening. As the disease progresses chronically, pulmonary nodules can evolve into masses or airspace consolidation. In some cases, calcification may be observed in the parenchymal portion of the lesion. The center of the air cavity consolidation contains low-density areas, often with cavity formation. Multiple thin-walled, smooth, wall-less nodules may be present. Additionally, hilar and mediastinal lymph nodes can show enhancement. Bronchoscopy plays a valuable role in the diagnosis of pulmonary actinomycetes. Based on bronchoscopic features, it can be classified into 5 types: occult, suppurative necrosis, endoluminal mass, organic erosion, and mixed phenotypes.^[27]^

Among them, the sulfur-like particles observed in the lumen of bronchial tubes in affected lesions during bronchoscopy have a significant value in suggesting an infection by actinomycetes. The examination of bronchial lavage fluid and alveolar lavage fluid through bacterial culture can also be useful; however, actinomycetes, being anaerobic bacteria, tend to have slow growth. They require cultivation in an anaerobic environment, with demanding cultivation conditions and high technical requirements. Additionally, the presence of symbiotic microorganisms in anaerobic environments and contamination from other microorganisms can impede the growth of actinomycetes. Consequently, the positivity rate of sputum culture is generally low.^[28]^ As a result, the diagnosis of most cases of pulmonary actinomycosis is achieved through transbronchoscopic lung biopsy, percutaneous lung puncture, or surgical excision to obtain tissue for pathological examination.^[29]^

Penicillin G is the most effective agent for treating pulmonary Actinomyces with an appropriately large dose (1800–24 million U/d), a prolonged treatment duration, and a daily intravenous infusion of penicillin for 2 to 6 weeks after diagnosis. This should be followed by oral penicillin V (or amoxicillin) treatment for 6 to 12 months.^[26]^ In cases where there is an allergy to penicillin or if the treatment is not effective, alternative antibiotics such as streptomycin, sulfanilamide, erythromycin, lincomycin, tetracycline, and cephalosporins can be considered. For chest wall abscesses or empyemas, it is necessary to close and drain the area. Additionally, surgical removal may be required for incurable actinomycete lung granulomas, fibrosis, bronchiectasis, chest wall or rib lesions, fistulas, and other related conditions.

## 4. Conclusion

Cases of pulmonary Actinomyces infection secondary to PS are rare.^[30]^ PS exhibits similar clinical features to pulmonary actinomyces, including multiple episodes of pneumonia and nonspecific imaging findings. When chest imaging indicates a basal segment of the lower lobe, a long-standing and incurable lesion adjacent to the spine, it is crucial to consider the possibility of PS. In such cases, performing chest-enhanced CT and 3-D reconstruction of the pulmonary vasculature promptly is necessary to confirm the diagnosis. Pulmonary actinomycetes infection lacks specific clinical manifestations. However, when imaging shows mass-like consolidation, cystic lesions, liquefactive necrosis, and pneumonia-like changes, a more typical sign known as the “air sign” may appear. Additionally, the presence of sulfur-like particle changes during tracheoscopy may indicate pulmonary actinomycetes infection. In such cases, it is important to perform a prompt biopsy to obtain a definite diagnosis and avoid missing or misdiagnosing the condition. In this case, the diagnosis of pulmonary sequestration was confirmed through chest-enhanced CT and 3-dimensional reconstruction examination of the pulmonary vessels. The postsurgical pathological diagnosis revealed pulmonary actinomycetes infection. Although the postoperative specimen was not sent for tissue microbial culture examination, timely fixation with formaldehyde immersion was performed. The aspiration of actinomycetes into the oral cavity after alcohol consumption is believed to have caused the infection of the sequestered pulmonary lesion tissue. The patient received successful treatment through surgical intervention and postoperative penicillin administration.

## Acknowledgments

We appreciate the invaluable work done by the medical staff of the pathology department, including the nurses, and the radiologists.

## Author contributions

**Conceptualization:** Yun Wang.

**Data curation:** Yun Wang, Na-na Rao.

**Formal analysis:** Yun Wang, Na-na Rao.

**Software:** Yun Wang.

**Writing – original draft:** Yun Wang.

**Writing – review & editing:** Mubo Liu, Jiangrong Liao, Qian-yu Wang.

## References

[1] Zhang N Zeng Q Chen C Yu J Zhang X. Distribution, diagnosis, and treatment of pulmonary sequestration: report of 208 cases. J Pediatr Surg. 2019;54:1286–92. 30291025 10.1016/j.jpedsurg.2018.08.054

[2] Wani SA Mufti GN Bhat NA Baba AA. Pulmonary sequestration: early diagnosis and management. Case Rep Pediatr. 2015;2015:454860. 26273485 10.1155/2015/454860 PMC4529943

[3] Carter R. Pulmonary sequestration. Ann Thorac Surg. 1969;7:68–88. 4883836 10.1016/s0003-4975(10)66147-4

[4] Smith RA. A theory of the origin of intralobar sequestration of lung. Thorax. 1956;11:10–24. 13311855 10.1136/thx.11.1.10 PMC1019399

[5] Hall NJ Stanton MP. Long-term outcomes of congenital lung malformations. Semin Pediatr Surg. 2017;26:311–6. 29110827 10.1053/j.sempedsurg.2017.09.001

[6] Kim HJ Shin KE Park JS. Intralobar pulmonary sequestration with cystic degeneration mimicking a bronchogenic cyst in an elderly patient: A case report and literature review. Medicine (Baltimore). 2020;99:e19347. 32118772 10.1097/MD.0000000000019347 PMC7478580

[7] Ren S Yang L Xiao Y Tong Z Wang L Hu Y. Pulmonary sequestration in adult patients: a single-center retrospective study. Respir Res. 2023;24:13. 36635696 10.1186/s12931-023-02320-w PMC9837954

[8] Cong CV Ly TT Duc NM. Intralobar pulmonary sequestration supplied by vessel from the inferior vena cava: literature overview and case report. Radiol Case Rep. 2022;17:1345–53. 35242263 10.1016/j.radcr.2022.01.082 PMC8866838

[9] Gao R Jiang L Ren Z Zhou L. Intralobular pulmonary sequestration in the middle lobe supplied by a right internal mammary artery: a case report. BMC Pulm Med. 2022;22:286. 35883077 10.1186/s12890-022-02083-y PMC9327363

[10] Zavadovsky KV Ilyushenkova JN Vasiltseva OY. Intralobar sequestration associated with the coronary-pulmonary artery fistula from the system of the circumflex artery. Circ Cardiovasc Imaging. 2020;13:e010234. 32842752 10.1161/CIRCIMAGING.119.010234

[11] Xie D Xie H You X Chen C Jiang G. Pulmonary sequestration with aberrant arteries arising from the renal artery and the internal thoracic artery. Ann Thorac Surg. 2013;96:e131. 24182513 10.1016/j.athoracsur.2013.08.018

[12] Marwah R Nair JR Singal A Talwar I. 3D multidetector CT angiographic evaluation of intralobular bronchopulmonary sequestration. J Indian Assoc Pediatr Surg. 2010;15:39–41. 21180507 10.4103/0971-9261.69144 PMC2998671

[13] Walker CM Wu CC Gilman MD Godwin JD Shepard JO Abbott GF. The imaging spectrum of bronchopulmonary sequestration. Curr Probl Diagn Radiol. 2014;43:100–14. 24791614 10.1067/j.cpradiol.2014.01.005

[14] Robson VK Shieh HF Wilson JM Buchmiller TL. Non-operative management of extralobar pulmonary sequestration: a safe alternative to resection? Pediatr Surg Int. 2020;36:325–31. 31707604 10.1007/s00383-019-04590-2

[15] Li Q Xie D Sihoe A. Video-assisted thoracic surgery is associated with better short-term outcomes than open thoracotomy in adult patients with intralobar pulmonary sequestration. Interact Cardiovasc Thorac Surg. 2018;26:284–7. 29049780 10.1093/icvts/ivx311

[16] Avsenik J Štupnik T Popovič P. Endovascular embolization prior to surgical resection of symptomatic intralobar pulmonary sequestration in an adult. Eur J Radiol Open. 2015;3:12–5. 27069973 10.1016/j.ejro.2015.11.001 PMC4811849

[17] Zhao K Li W Kang C. Phylogenomics and evolutionary dynamics of the family Actinomycetaceae. Genome Biol Evol. 2014;6:2625–33. 25245410 10.1093/gbe/evu211 PMC4224338

[18] Lortorici S Burruano F Buzzanca ML. Cervico-facial actinomycosis: epidemiological and clinical comments. Am J Infect Dis J. 2008;4:204–8.

[19] Moghimi M Salentijn E Debets-Ossenkop Y Karagozoglu KH Forouzanfar T. Treatment of cervicofacial actinomycosis: a report of 19 cases and review of literature. Med Oral Patol Oral Cir Bucal. 2013;18:e627–32. 23722146 10.4317/medoral.19124 PMC3731091

[20] Mato N Oshikawa K Sakuma Y Sawai T Ohno S Sugiyama Y. Thoracic actinomycosis: clinical, radiological, and pathological findings in 11 cases. Nihon Kokyuki Gakkai Zasshi. 2003;41:514–20. Japanese. 14503335

[21] Ito T Yoshida T Sakuraba M Tanaka A Hamada K Ito A. Insufficient initial treatment but good recovery after diagnosis of pulmonary actinomycosis. Oxf Med Case Rep. 2019;12:510–2. 10.1093/omcr/omz123 PMC6937452 31908824

[22] Rupani A Amonkar G Deshpande J. Pulmonary actinomycosis masquerading as tuberculosis. Indian J Pathol Microbiol. 2009;52:438–9. 19679988 10.4103/0377-4929.55021

[23] Grzywa-Celińska A Emeryk-Maksymiuk J Szmygin-Milanowska K Czekajska-Chehab E Milanowski J. Pulmonary actinomycosis - the great imitator. Ann Agric Environ Med. 2017;25:211–2. 29936825 10.26444/aaem/75652

[24] Sun XF Wang P Liu HR Shi J-H. A retrospective study of pulmonary actinomycosis in a single institution in China. Chin Med J (Engl). 2015;128:1607–10. 26063362 10.4103/0366-6999.158316 PMC4733730

[25] Varshney MK Trikha V Khan SA. Actinomycosis or tuberculosis? A diagnostic dilemma. Scand J Infect Dis. 2006;38:378–81. 16709542 10.1080/00365540500447168

[26] Mabeza GF Macfarlane J. Pulmonary actinomycosis. Eur Respir J. 2003;21:545–51. 12662015 10.1183/09031936.03.00089103

[27] Zhang GZ Du JH Yuan WY. Study on bronchoscopic features and classification of pulmonary actinomycosis. China J Endosc. 2021;27:79–87. Chinese.

[28] Wang L Zhang H Wu D. Pulmonary lesions associated with sputum culture-positive actinomycetes: report of one case. Ann Transl Med. 2019;7:793. 32042809 10.21037/atm.2019.12.38 PMC6990027

[29] Hoca NT Berktaş MB Söyler Y Celep C Tanrikulu FB. Clinical features and treatment outcomes of pulmonary actinomycosis. Eur Rev Med Pharmacol Sci. 2022;26:8064–72. 36394726 10.26355/eurrev_202211_30160

[30] Chaves JJ Nieto FP Gómez-Gómez M Rodríguez DF García-Concha D Parra-Medina R. Pulmonary sequestration associated with actinomycosis: a case report. Antibiotics (Basel). 2020;9:687. 33050328 10.3390/antibiotics9100687 PMC7599791

